# Intraventricular infusion of hyperosmolar dextran induces hydrocephalus: a novel animal model of hydrocephalus

**DOI:** 10.1186/1743-8454-6-16

**Published:** 2009-12-11

**Authors:** Satish Krishnamurthy, Jie Li, Lonni Schultz, James P McAllister

**Affiliations:** 1 Department of Neurosurgery, Upstate Medical University, Syracuse, NY 13210, USA; 2 Department of Biostatistics and Research Epidemiology, Henry Ford Hospital, 2799, West Grand Blvd, K 11, Detroit MI 48202, USA; 3 Department of Neurosurgery, Division of Pediatric Neurosurgery, 175 N Medical Drive East, Salt Lake City, UT 84132, USA

## Abstract

**Background:**

Popular circulation theory of hydrocephalus assumes that the brain is impermeable to cerebrospinal fluid (CSF), and is therefore incapable of absorbing the CSF accumulating within the ventricles. However, the brain parenchyma is permeable to water due to the presence of specific ion channels as well as aquaporin channels. Thus, the movement of water into and out of the ventricles may be determined by the osmotic load of the CSF. If osmotic load determines the aqueous content of CSF in this manner, it is reasonable to hypothesize that hydrocephalus may be precipitated by pathologies and/or insults that produce sustained elevations of osmotic content within the ventricles.

**Methods:**

We investigated this hypothesis by manipulating the osmotic content of CSF and assaying the development of hydrocephalus in the rat brain. This was achieved by continuously infusing artificial CSF (negative control; group I), fibroblast growth factor (FGF2) solution (positive control; group II) and hyperosmotic dextran solutions (10 KD and 40 KD as experimental solutions: groups III and IV) for 12 days at 0.5 μL/h. The osmolality of the fluid infused was 307, 664, 337 and 328 mOsm/L in Groups I, II, III and IV, respectively. Magnetic resonance imaging (MRI) was used to evaluate the ventricular volumes. Analysis of variance (ANOVA) with pairwise group comparisons was done to assess the differences in ventricular volumes among the four groups.

**Results:**

Group I had no hydrocephalus. Group II, group III and group IV animals exhibited significant enlargement of the ventricles (hydrocephalus) compared to group I. There was no statistically significant difference in the size of the ventricles between groups II, III and IV. None of the animals with hydrocephalus had obstruction of the aqueduct or other parts of CSF pathways on MRI.

**Conclusion:**

Infusing hyperosmolar solutions of dextran, or FGF into the ventricles chronically, resulted in ventricular enlargement. These solutions increase the osmotic load in the ventricles. Water influx (through the choroid plexus CSF secretion and/or through the brain) into the ventricles to normalize this osmotic gradient results in hydrocephalus. We need to revise the popular theory of how fluid accumulates in the ventricles at least in some forms of hydrocephalus.

## Background

Hydrocephalus is a brain disorder which manifests as an abnormal accumulation of cerebrospinal fluid (CSF) in the ventricles. It affects patients of all ages and is associated with either congenital or acquired factors, including brain trauma, infection, tumors, hemorrhage and stroke. Hydrocephalus is generally harmful to brain tissues and can result in both structural damage and cognitive impairment [1]. In the United States, hydrocephalus accounts for approximately 70,000 hospital admissions annually, with associated health care costs estimated at over two billion dollars [2].

Current strategies for diagnosing and treating hydrocephalus are based on our limited understanding of its underlying pathogenesis. Circulation theory states that CSF, produced mainly by the choroid plexus, flows through the ventricles along specific pathways to the subarachnoid space, where it is absorbed through Pacchionian granulations into venous sinuses. Obstruction of any part of this CSF circulation causes an abnormal accumulation of CSF in the ventricles resulting in hydrocephalus [3-5]. Further, popular understanding does not consider alternate pathways of CSF absorption especially through nasal lymphatics [6]. Diagnostic techniques are therefore focused primarily on detecting increases in ventricle size and blockages of CSF circulation, whereas treatment consists of medical and/or surgical interventions aimed at preventing the buildup of CSF and the associated elevation of intracranial pressure. However, hydrocephalus commonly occurs in the absence of demonstrable obstructions of CSF circulation and/or increases in intracranial pressure [7-11].

One of the fundamental assumptions of circulation theory is that the brain parenchyma is impermeable to CSF, and is therefore incapable of absorbing the CSF accumulating within the ventricles. However, the brain parenchyma is permeable to water [12]. The molecular basis of this permeability involves specific ion channels which permit water movement with ions as well as aquaporin channels, which permit the free movement of water without changing the ionic environment [13]. Aquaporin channels are membrane proteins that have an ion trap and allow movement of water without allowing movement of ions. Aquaporin 4 (AQP4) channels are found in the ependymal cells lining the lateral ventricles, and on the end feet of astrocytes that contact microvessels in the periventricular white matter and the subpial region of the cerebral cortex [14]. The distribution of AQP4 within the brain suggests that the aqueous content of CSF may be increased or decreased as water moves through the brain parenchyma between the ventricles and vascular system. Thus, the movement of water into and out of the ventricles may be determined by the osmotic load of the CSF, which is a function of the presence, type, and amount of non-diffusible macromolecules.

If osmotic load determines the aqueous content of CSF in this manner, it is reasonable to hypothesize that hydrocephalus may be precipitated by pathologies and/or insults that produce sustained elevations of osmolality within the ventricles. Our experiments were designed to test this hypothesis by experimentally manipulating the osmotic content of CSF and assaying the development of hydrocephalus in the normal rat brain. This was achieved by continuously infusing solutions of different macromolecules with different osmolarities into the lateral ventricle for 12 days, and assaying the associated enlargement of the lateral and third ventricles [15]. The resulting hydrocephalus evolved rapidly and reproducibly and was monitored using magnetic resonance imaging (MRI).

## Methods

### Animals and experimental groups

Adult female Sprague-Dawley rats (220-250 g; Harlan, Indianapolis, Indiana, USA) were housed in the animal care facility during a 12-h light/dark cycle throughout the protocol. All efforts were made to minimize the suffering and the number of animals used. Animal care and surgical procedures were carried out in accordance with protocol approved by the Institutional Animal Care and Use Committee at Wayne State University.

### Cannula implantation

After achieving a surgical plane of anesthesia (loss of corneal, pupillary, and limb withdrawal reflexes) with a dose of 87 mg/kg ketamine plus 13 mg/kg xylazine administered intraperitoneally, rats (220-250 g body weight) were secured in a stereotaxic head frame. The eyes were moistened by the application of eye ointment. The skin was incised along the midline and the dorsal surface of the skull exposed. With a variable speed drill, a 1.5 mm diameter craniotomy was performed over the right cerebral hemisphere 0.9 mm caudal and 1.2 mm lateral to Bregma leaving the dura intact. A stainless steel needle (27 gauge) was advanced through the opening to a depth of 3.6 mm below the level of the dura at 90° to the skull surface. These coordinates place the tip of needle in the frontal horn of the lateral ventricle. After slowly pulling out the needle, a customized microcatheter that was connected to an Alzet osmotic minipump was inserted through the needle track to reach the lateral ventricle. The microcatheter had the same diameter as a 27 gauge needle and was made by heating polyethylene tubing, PE 50. The microcatheter was fixed in place by covering a piece of Surgicel (ETHICON, Inc. Somerville, USA) with cyanoacrylate adhesive (DURECT Corporation, Cupertino, USA). One or two sutures were placed with the connecting tissue around the catheter for further security. A minipump was then inserted into a subcutaneous pocket on the back in the mid-scapular region. The skin incision was closed with monofilament nylon suture. The Alzet osmotic minipump (Model 2002, DURECT) was primed with the solution prior to implanting. The osmolality of solutions was measured by osmometer before loading into the pump. The solution was infused at 1 μg per day (0.5 μL/h) at a concentration of 0.083 μg/μL, apart from the hypertonic saline which had a concentration of 58.44 μg/μL.

After 2-3 hours, once the animal had regained consciousness and had demonstrated normal feeding and drinking behavior, it was returned to its cage and monitored periodically for the duration of the experiments. Animals were sacrificed by cardiac perfusion with saline followed by fixative buffer after the 12 days of infusion and after the final MRI scan and brain tissue was saved for further histological analysis.

### Lateral ventricle infusions

Rats were randomly divided into 4 primary groups.

Group I: Artificial cerebrospinal fluid (ACSF, n = 6): This group served as negative control since artificial CSF has the same osmolality as the CSF and is not expected to result in hydrocephalus. The ACSF was prepared according to a previously published method [16] and the osmolality was 307 mOsm/L, measured using Micro Freezing Point Osmometer (Model 3300; Advanced Instruments, Inc., Norwood, MA, USA)

Group II: Fibroblast growth factor 2 (FGF-2, n = 4): This group served as a positive control. The concentration of FGF-2 (R&D systems, Minneapolis, USA) was 0.083 μg/μL and the vehicle used was 20 mM Tris-HCl and 1.0 M NaCl (pH = 7.0) [15]. The osmolality of the FGF-2 solution was 664 mOsm/L.

Group III: 10 KD dextran (n = 8): This solution was hyperosmotic and was expected to result in hydrocephalus if our hypothesis was correct. The concentration of the 10 KD dextran in the solution was 0.083 μg/μL and the osmolality was 337 mOsm/L.

Group IV: 40 KD dextran (n = 8): This solution was hyperosmotic and was also expected to result in hydrocephalus if our hypothesis was correct. The concentration of the 40 KD was 0.083 μg/μL and the osmolality was 328 mOsm/L.

Both the protein (FGF-2) as well as the vehicle used (1 Molar saline) contributed to the osmolality of FGF-2 solution (664 mOsm/L). Based on our hypothesis, both of these hyperosmotic solutions should result in ventricular enlargement. Hence, we investigated the effect of FGF-2 and hypertonic saline separately and these groups were not included with the primary groups due to the small number of animals in each group.

Group V: FGF-2 solution with normal saline [FGF2(NS)], (n = 2): We used dialysis tubing (10 KD) to dialyse this FGF-2 against sterile saline over night and adjusted the concentration to 0.083 μg/μL and re-measured the osmolality, which was 326 mOsm/L. We used this solution to investigate the effect of FGF-2 without its hyperosmolar vehicle.

Group VI: Hypertonic saline solution (n = 3): This solution was prepared at a concentration of 58.44 μg/μL and the osmolality was 910 mOsm/L. This solution was expected to result in hydrocephalus if our hypothesis was correct.

### MRI scans and ventricular volume calculation

Anatomical MRI images were taken in the Magnetic Resonance Research Facility at Wayne State University. MRI scans were performed after 12 days of infusion. The animal was anesthetized with ketamine and xylazine administered intraperitoneally as described previously and was placed into the MRI scanner. All MRI measurements were performed on a 4.7-T horizontal-bore magnetic resonance spectrometer (Bruker AVANCE) with an 11.6-cm-bore actively shielded gradient coil set capable of producing a magnetic field gradient of up to 250 mT/m. A whole-body birdcage radiofrequency (RF) coil (inner diameter, 72 mm) was used as the transmitter for homogeneous RF excitation, and a surface coil (30 mm diameter) was used as the receiver, with active RF decoupling to avoid signal interference. Coronal axial T2-weighted images (Rapid Acquisition Relaxation Enhanced or RARE sequences which minimize motion related artifacts) were acquired using the following parameters: TR 5 sec, TE(eff) 57 ms, FOV 32 × 32 mm^2^, 1 signal average, 1 mm slice thickness, interleaved 24 slices, TA 2m40s.

In order to calculate the ventricular volumes, we used the semi-automated method as reported by previous studies [17,18]. The edge of the ventricles (region of interest) for all transverse slices was manually outlined, and the surface area was determined by counting the number of pixels enclosed by the edge automatically by the software. Segmental volume was calculated by multiplying slice area by slice thickness, and total T2-weighted MRI volume was determined by summation of the segmental volumes. Internally developed MR SPIN (Signal Processing in MR) software written in Visual C++ on the Microsoft Windows platform was used for MRI and ventricular volume calculation. Some animals died during the experiment (n = 2) but were not included in the analysis.

### Statistical methods

ANOVA with pair-wise group comparisons was done to assess the differences in ventricular volumes among the four groups. A log transformation was used to reduce the variability across the groups. A *p *value of less than 0.05 was considered to be significant.

## Results

**Figure 1:**
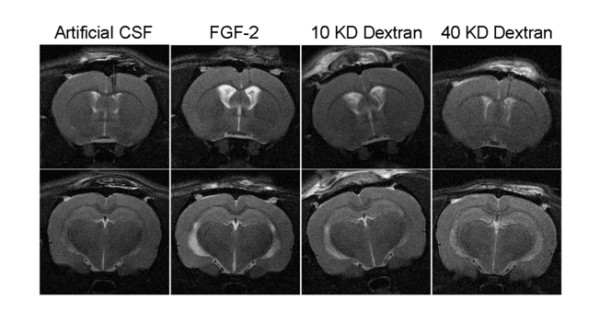
**Representative examples of MRI images of animals after 12 days of infusion of artificial CSF (negative control), FGF-2 (positive control), 10 KD and 40 KD dextran (experimental) solutions**. Note that the ventriculomegaly produced by FGF-2, 10 KD and 40 KD dextrans were similar.

**Figure 2:**
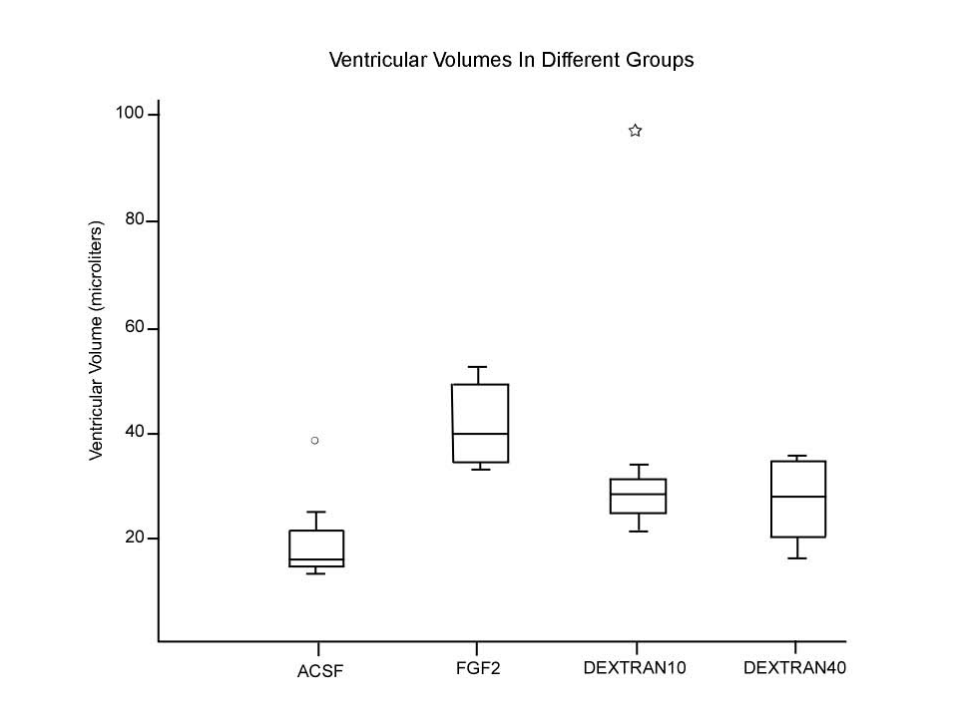
**Box plot showing the ventricular volumes in μL for the different infusion groups**. The center line of the boxplot is the median value with the upper and the lower margins of the box representing the upper quartile (75^th ^percentile) and the lower quartile representing (25^th ^percentile). The upper and lower fences represent the value equal to 1.5 times the difference between the lower and upper quartiles (interquartile ratio). The outliers are represented by a mark outside the box plot. Note that all the infusions resulted in enlarged ventricles except for ACSF, and were significantly different from the ACSF group (I vs II *p *= 0.002, I vs III *p *= 0.009, and I vs IV *p *= 0.023). Note: Two outlying symbols represent ventricular size of animals in the ACSF and 10 KD dextran group that were much larger than the rest of the group.

Animals in group I (ACSF) had no hydrocephalus. Group II (FGF2), group III (10 KD) and group IV (40 KD) animals exhibited significant enlargement of the ventricles (hydrocephalus) compared to group I (I vs II *p *= 0.002, I vs III *p *= 0.009, and I vs IV *p *= 0.023; Figures 1 and 2). There was no statistically significant difference in the size of the ventricles between any of the groups II, III and IV. The ventricular volumes for animals in all groups are listed in the Table 1. The osmolality of the fluid infused was 307 mOsm/L(Group I:ACSF), 337 mOsm/L (Group III: 10 KD)and 328 mOsm/L (Group IV; 40 KD). Additional two sample t-tests were done to compare group I (ACSF) to group V (FGF2 with normal saline) and VI (Hypertonic saline). Group V FGF-2-infused animals had significant hydrocephalus compared to Group I (mean (SD) 42.4 (1.5) vs 23.6 (8.6) μL; *p *= 0.04). Group VI hypertonic saline-infused animals also showed a trend to induced hydrocephalus when compared to Group I (mean (SD) 33.9 (3.9) μL vs 23.6 (8.6) μL) although it was not significant.

**Table 1:** Ventricular volumes (μL) in animals for the different infusion groups.

	Group I ACSF	Group II FGF2	Group III 10 KD Dextran	Group IV 40 KD Dextran	Group V FGF2 (NS)	Group VI Hypertonic Saline
	17.95	51.52	40.82	38.02	41.40	29.64

	16.43	33.17	43.55	34.60	43.46	37.29

	25.85	43.16	31.18	20.33		34.71

	39.58	34.82	35.74	25.95		

	18.39		30.77	38.10		

	23.48		41.78	39.00		

			96.54	36.67		

			24.81	35.53		

**Mean**	23.61	40.67	43.15	33.53	42.43	33.88

**Median**	20.94	38.99	38.28	36.10	42.43	34.71

**SD**	8.61	8.45	22.51	6.74	1.46	3.89

**Figure 3:**
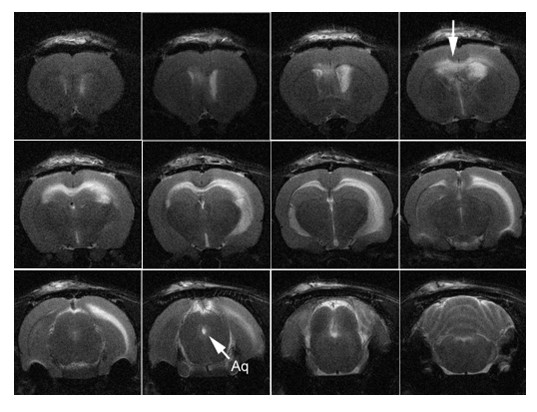
**T2-weighted MRI of animal with hydrocephalus induced by 10 KD dextran**. Note the periventricular edema (arrow top row last figure from the left) in the corpus callosum and external capsule and the patent cerebral aqueduct (arrow labeled Aq). Note that the ventricular enlargement was asymmetric with the larger ventricle on the side of infusion.

Ventriculomegaly was moderate and was consistent throughout the lateral and the third ventricles but not apparent in the fourth ventricle. The ventricular enlargement was asymmetric in some animals with the larger ventricle on the side of infusion. There were two animals which had ventricular size that were much larger than the rest of the group. One of them was in the ACSF group and the other was in the 10 KD group. This was probably due to variability in the size of the ventricles prior to infusion, rather than an effect of the infusing solutions. We do not know the exact reason in this set of experiments as we did not perform MRI scans prior to infusing the solutions. However, in the experiments that we have done since, we have seen such variability in the pre-infusion scans. None of the animals with hydrocephalus had obstruction of the aqueduct or other parts of CSF pathways on MRI (Figure 3).

## Discussion

We hypothesized that osmotic load in the ventricles determines the aqueous content of CSF. Our experiments were designed to test this hypothesis by experimentally manipulating the osmotic load of CSF and assaying the development of hydrocephalus in the normal rat brain. This was achieved by continuously infusing hyperosmolar solutions of FGF-2, 10 KD and 40 KD dextrans into the lateral ventricle for 12 days. There was significant enlargement of ventricles on the MRI in all these groups compared to infusing iso-osmolar ACSF. The MRI scan did not show any obstruction of the aqueduct or other parts of the CSF pathways on MRI.

Popular CSF circulation theory explaining the genesis of hydrocephalus fails to consider various clinical observations and also fails to consider some experimental findings [19-27]. In addition, current diagnostic and therapeutic strategies that are based on this framework of thinking fall short in helping to treat patients with hydrocephalus effectively. One such clinical example is the current algorithm used for diagnosing hydrocephalus. Hydrocephalus is diagnosed by computed tomography (CT) examination or MRI scan and correlated with clinical symptoms, to determine treatment. Although radiological diagnosis is effective for most patients, there are several situations that pose problems. The size of the ventricles alone does not determine whether a patient has symptoms of hydrocephalus [28]. Further, ventricle size has no relationship to the pressure inside the ventricle; examples include pseudotumor cerebri (small ventricles with high pressure), normal pressure hydrocephalus (NPH, large ventricles with normal pressure), and stiff non-compliant ventricles (small ventricles with low, normal or high pressures). Pressure-volume relationships have been used to evaluate the need for treatment of hydrocephalus from different conditions [29,30]. Intracranial pressure recording is more helpful but does not always predict who will benefit from surgery to relieve hydrocephalus [31]. In fact, a recent paper on treatment of NPH highlights the limitations of current diagnostic methods in diagnosing hydrocephalus and predicting outcomes following surgery [32]. Clinical practice becomes subjective and varies widely as a consequence of uncertainties in interpretation of clinical and radiological data even among experts in the field. Clinical decision making would definitely benefit from a better understanding of pathophysiology of hydrocephalus. This is the motivation for revisiting the current concept of pathogenesis of hydrocephalus.

There is circumstantial evidence to the role played by the osmotic gradients. It is well known clinically that osmotic gradients play a role in the brain tissues (excluding the ventricular space) in normal and abnormal states. For example, in brain edema, osmotic diuretics like mannitol are used intravenously to draw water away from the extracellular space of the brain. Brain swelling can result in conditions where there is hyponatremia which permits water movement into the tissues of the brain resulting in brain edema [33]. Clinically, high protein levels in the CSF have been detected in hydrocephalus irrespective of radiologic obstruction to the CSF pathways. Higher levels of thrombopoietin [34], ferritin [35], nerve growth factor[36], chondroitin sulfate proteoglycan [37] transforming growth factor β1 [38,39], transforming growth factor β2 [37], S-100 protein [40,41], and vascular endothelial growth factor [42] have been found in ventricular CSF in patients with hydrocephalus resulting from intraventricular hemorrhage. Even in patients without any intraventricular blood, elevated proteins have been found in ventricular CSF in hydrocephalus resulting from intracranial schwannomas [7], a few cases of spinal schwannomas [8,9], and in about 4% of patients with Guillain-Barre' syndrome [10,11]. In a review of potential biomarkers for chronic hydrocephalus, Tarnaris *et al *concluded that tumor necrosis factor, tau protein, lactate, sulfatide and neurofilament triple protein are elevated in chronic hydrocephalus to make them the most promising CSF markers [43]. In addition to proteins, increased levels of lactate [43,44] and lactic dehydrogenase (LDH) [45] have been found in hydrocephalus. Increased levels of ions (calcium, magnesium and phosphate) found in congenital hydrocephalus correlated with elevated protein levels [46]. An extensive summary of changes in the composition of CSF in hydrocephalus was reviewed by Del Bigio [47]. It is unclear whether these changes in proteins or peptides are a cause or a consequence of hydrocephalus.

The importance of the role played by excess macromolecules in the ventricle in hydrocephalus is strengthened by the relief of hydrocephalus in situations that decrease the amount of macromolecules in the CSF. Decreasing levels of protein and blood products with removal of CSF through an Ommaya ventricular reservoir is associated with resolution of hydrocephalus in about 17% of neonates with intraventricular hemorrhage [48,49]. The elimination of blood and blood products decreases the incidence of hydrocephalus due to aneurysmal subarachnoid hemorrhage [50]. One explanation of how elimination of macromolecules results in relief of hydrocephalus is the decrease in the osmotic load in addition to reduction of their biological effect on the brain and CSF secretion.

In addition to the clinical evidence, some experimental evidence suggests that osmolality plays a role in the genesis of hydrocephalus, although there have been no studies focusing primarily on this mechanism. Wald *et al *found that increasing the ventricular fluid osmolality in a perfusate increased the volume of CSF produced [51] and increasing the serum osmolality decreased CSF production in normal cats [52]. These experiments led the authors to conclude that CSF production is influenced by the osmotic gradient between the serum and the ventricular CSF. In other experiments, infusion of protein (FGF-2) into the lateral ventricles of experimental animals caused dilatation of the ventricles [15].

The strongest argument against circulation theory comes from experiments focusing on the development of ventricles in embryos. It is well known that brain ventricles are a highly conserved system of cavities that form early during brain morphogenesis in vertebrates and are required for normal brain function [53]. In a series of elegantly done experiments, Lowery and Sive were able to show that initial ventricle expansion occurs independently of circulation and is related to cellular proliferation in zebrafish embryos [54]. Although onset of circulation contributed to continued expansion of the ventricles, ventricular expansion occurred in silent heart mutant zebrafish embryos which do not have a beating heart [54].

The neural tube is a single cell layer tube that is permeable to water. Expansion of this tube involves changes in the osmolality of the neural tube fluid. As another example, Alonso *et al*, during an investigation of the underlying mechanism of neural tube expansion in chick embryos found that increasing the neural tube fluid osmolality resulted in hydrocephalus [53]. These studies clearly suggest that osmolality of the fluid in the ventricles plays a role in the regulation of the size of the ventricles at least in the embryonic stage.

## Conclusion

Continuous infusion of large molecules such as FGF and dextran into the lateral ventricles resulted in ventricular enlargement (hydrocephalus). It is concluded that increasing the osmotic load results in water influx (through choroid plexus CSF secretion and/or through the brain) into the ventricles to normalize the osmotic gradient. The popular theory of how fluid accumulates in the ventricles may need to be revised, at least in some forms of hydrocephalus. It is important to note that our concept does not affect the pressure, volume and compliance changes seen in hydrocephalus.

## Competing interests

The authors declare that they have no competing interests.

## Authors' contributions

SK designed the study, was primary investigator and primary author. J L performed the experiments, obtained the MRI scan data, assisted in designing the study and writing the article. P M helped design and fund the study and assisted in writing the article. LS performed statistical analysis and helped write the article.

All authors have read and approved the final version of the manuscript.
